# Association of hormone replacement therapy with risk of gastric cancer: a systematic review and meta-analysis

**DOI:** 10.1038/s41598-022-17345-2

**Published:** 2022-07-29

**Authors:** Yeu-Chai Jang, Chi Yan Leung, Hsi-Lan Huang

**Affiliations:** 1 Wan Fang Hospital, Taipei Medical University, Taipei, Taiwan; 2 Department of Global Health Policy, Graduate School of Medicine, The University of 7-3-1 Hongo, Bunkyo-ku, Tokyo, 113-0033 Japan

**Keywords:** Cancer prevention, Preventive medicine

## Abstract

Hormone replacement therapy (HRT) is widely used to relieve menopausal symptoms; however, it remains unclear whether the use of HRT was associated with gastric cancer. We conducted a systematic review and meta-analysis to synthesize available evidence. This study followed the PRISMA guideline to report meta-analysis. PubMed, Embase, and Cochrane library were searched from conception through 23 February 2022. Eligible studies reporting risk of gastric cancer after HRT were screened and accessed by two independent reviewers. Random-effects meta-analysis was used to calculate pooled risk estimate as relative risk (RR, 95% CI). Pre-established review protocol was registered in PROSPERO (CRD42021281260). Among the 1095 articles identified, we included 11 studies with 1,919,089 women in this meta-analysis. The combined risk estimate (RR, 0.72; 95% CI 0.64–0.81; *I*^*2*^ = 2%) indicated that the use of HRT was associated with a 28% reduction in risk of gastric cancer compared with those who had no HRT exposure. The narrow prediction interval (0.62–0.84) for gastric cancer risk suggested a low between-study variance. In subgroup analysis defined by HRT formulation, there were reduction in risks of gastric cancer after the use of estrogen-only therapy (Pooled RR, 0.63; 95% CI 0.51–0.77, *I*^*2*^ = 0%) and estrogen-progestin therapy (Pooled RR, 0.70; 95% CI 0.57–0.87; *I*^2^ = 0%), as compared with non-users. In this systematic review and meta-analysis, the use of HRT was associated with a reduced gastric cancer risk regardless of HRT formulation. Further investigations are warranted to confirm underlying mechanisms.

## Introduction

Gastric cancer was the sixth commonly diagnosed cancer globally, responsible for 768,793 cancer deaths in 2020^1^. Albeit recent efforts shed light on the reduction of gastric cancer burden^1–4^ and the present decreasing trend in incidence rate has been projected to continue^5^, it was estimated that there will be 1,596,319 gastric cancer cases in 2035^6^.

Hormone replacement therapy (HRT), including various estrogen-only and estrogen–progestin combined regimens, is widely prescribed to relieve menopausal symptoms, such as night sweats, hot flushes and mood swings^7^. The use of HRT was also linked to osteoporosis prevention^8^. Previous studies suggested that the use of HRT may be associated with an increased risk of breast cancer and ovarian cancer^9,10^. On the other hand, several studies have shown a reduction in the risk of esophageal cancer and colorectal cancer in women with HRT^11–15^.

In 2012, a meta-analysis reported an inverse association between the overall use of HRT and gastric cancer risk^16^. Since then, more studies have been published but the results were inconsistent^17–19^. In addition, different hormone combinations are available, resulting in concerns about benefits and harms of specific formulations. However, little is known about the associations of different formulations of HRT with the risk of gastric cancer. Achieving individualised treatment and making informed decisions among women with menopausal symptoms require clear and consistent evidence, and therefore, further assessment of gastric cancer risk associated with specific types of HRT are needed. In this study, we sought to perform a systematic review and meta-analysis to summarize the association of HRT use with gastric cancer incidence.

## Methods

We performed this systematic review and meta-analysis in accordance with the pre-established review protocol registered in PROSPERO (CRD42021281260). This study followed the Preferred Reporting Items for Systematic Reviews and Meta-Analyses (PRISMA) guidelines and Meta-analysis of Observational Studies in Epidemiology (MOOSE) reporting guideline (see Supplementary file 1: Table S1–2).

### Search strategy and selection criteria

Two reviewers (CYL and HLH) systematically searched human studies reporting the association of hormone replacement therapy and the risk of gastric cancer on the following electronic databases from inception to 23 February 2022: PubMed, Embase, and Cochrane library (the detailed search strategy is described in Supplementary file 1: Table S3–5). We placed no language restrictions. The titles and abstracts of retrieved articles were independently screened by two reviewers (CYL and HLH). Relevant articles were identified using keywords and Mesh terms relating to hormone replacement therapy and gastric cancer. We also reviewed the reference lists of all included studies. For articles in languages other than English, we consulted native speakers for translation. Any disagreement was resolved by consensus.

We included studies if they met the prespecificed criteria: (1) published original researches in human who had no prior cancer diagnosis with data on the use of hormone replacement therapy in relation to the risk of gastric cancer; and (2) study designs were randomised controlled trials, cohort studies, or case–control studies. Exclusion criteria included studies assessing hormone replacement therapy and cancer mortality; and cross-sectional studies, reviews, case reports, letters, and animal studies. We applied no restrictions on the route of hormone replacement therapy administration. The primary outcome was risk of gastric cancer after hormone replacement therapy. In this review, only studies that provided hazard ratio (HR), relative risk (RR), or odds ratio (OR) with 95% confidence intervals (CIs); or provide sufficient data that would allow the risk estimate to be calculated were eligible for inclusion.

### Data extraction and quality assessment

Two reviewers (CYL and HLH) independently reviewed all identified articles to extract the following data using a standardised observation form: name of first author, publication year, country, study design, study period, age, sample size, information on hormone replacement therapy, numbers of outcomes, adjustment, and risk estimates. A third reviewer (YCJ) performed verification. The Newcastle–Ottawa Quality Assessment Scale (NOS) was used to assess the methodological quality of all included studies^20^.

### Data synthesis and analysis

In this study, pooled estimates of relative risks were synthesised using random-effects meta-analysis, considering both within- and between-study variation. Methodological and clinical heterogeneity was assessed by *I*^2^ statistic to quantify the percentage of variation attributable to between-study heterogeneity. The *I*^2^ was categorised as low (≤ 50%), moderate (51–75%), and high (> 75%) heterogeneity^21^. Predictive intervals describing the heterogeneity in random-effects meta-analysis were estimated to inform the potential future treatment effect in 95% of all populations^22^. Visual inspection of Begg funnel plot and Egger test were performed to evaluate potential publication bias and small-study effects^23^. Trim and fill method was performed if publication bias existed^24^. Subgroup analyses were performed based on different types of HRT (estrogen-only HRT or estrogen-progestin HRT) for the risk of gastric cancer. Additional sensitivity analysis was performed to assess the robustness of the primary meta-analysis by using a fixed effect model to rerun the analysis. To assess the impact of individual studies on primary analysis, we conducted leave-one-out meta-analysis by omitting one study at a time^25^. In the present study, exact *p* values are provided unless p < 0.0001. Data were analysed using STATA version 16.1 (College Station, TX, USA).

## Results

### Literature search

**Figure 1:**
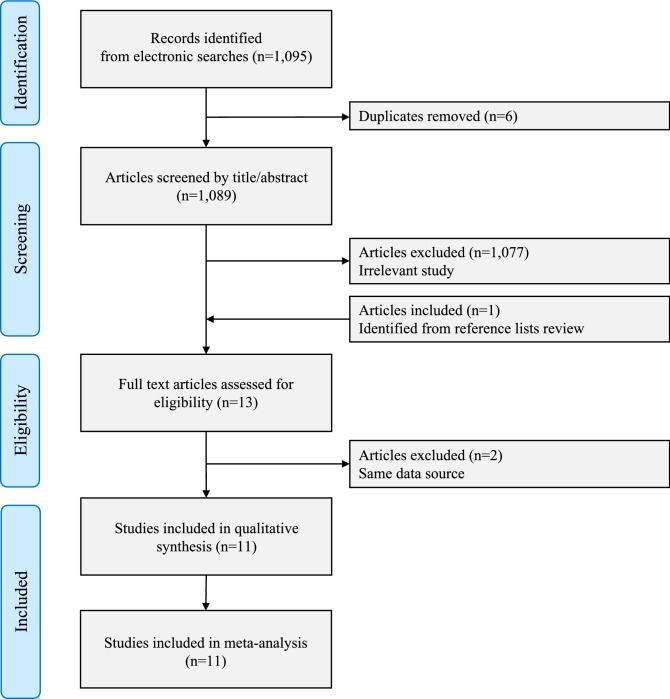
Flow diagram of study selection. *n* number.

As shown in Fig. 1, 1095 potentially relevant articles were identified after initial literature search from PubMed, Embase and Cochrane library. Among the 1095 articles, 1089 remained after removing six duplicated articles. We then excluded 1077 irrelevant articles after the review of titles and abstracts. One article was included from the references lists^26^, leaving 13 articles for full-text review. An additional two studies were excluded because of overlapping data sources^27,28^. Of studies with the same data source, the study with longer duration was included in the analysis. Finally, 11 studies were included in qualitative assessment and meta-analysis^17–19,26,29–35^.

### Characteristics of included studies

Table S6 shows the key characteristics of the included studies. In total, seven cohort studies^17,18,26,29–32^, three case–control studies^19,34,35^, and one nested case–control study^33^ were eligible for inclusion in the meta-analysis. The studies were published between 2003 and 2021 and included cohorts from 16 countries (Canada, China, Denmark, France, Germany, Greece, Italy, Japan, Korea, Netherlands, Norway, Singapore, Spain, Sweden, United Kingdom, and The United States). The participant number ranged from 652 to 1,160,351, which resulted in a total of 1,919,089 participants included in this meta-analysis. Study quality scores, assessed by NOS, were between low and high. Table S7–8 presents the study quality scores (see Supplementary file 1).

### Meta-analysis and subgroup analysis

**Figure 2:**
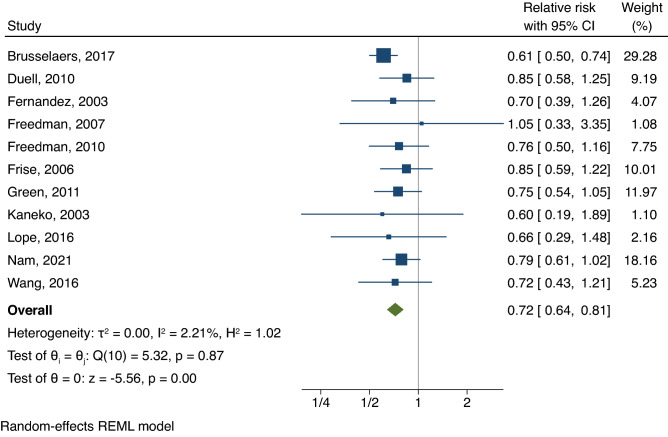
Summary of pooled risk estimates. The association between HRT use and risk of gastric cancer. *CI* confidence intervals, *HRT* hormone replacement therapy.

**Figure 3:**
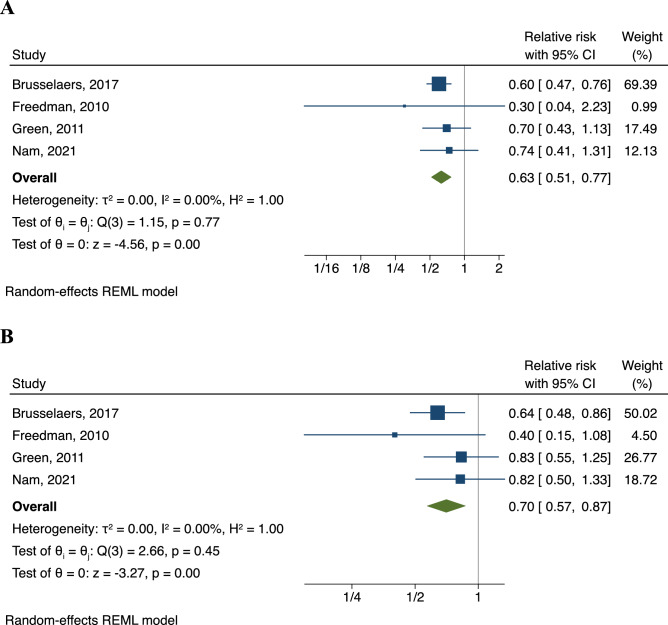
Subgroup analyses. The association of the use of (**A**) estrogen-only HRT and (**B**) combined estrogen-progestin therapy with gastric cancer risk. *RR* relative risk, *CI* confidence intervals, *HRT* hormone replacement therapy.

The pooled risk estimate from 11 studies with 1,919,089 participants in the meta-analysis showed that, as compared with non-users, individuals who received HRT had a 28% lower risk (RR, 0.72; 95% CI 0.64–0.81; *I*^2^ = 2%) of gastric cancer (Fig. 2). After accounting for between-study variance, the prediction interval (0.62–0.84) indicated that a future study is likely to yield an inverse association between HRT and risk of gastric cancer (Figure S1). In subgroup analyses defined by the type of HRT, the pooled RR was 0.63 (95% CI 0.51–0.77, *I*^2^ = 0%) for individuals who had estrogen-only HRT, comparing with non-users (Fig. 3A). There was a 30% lower risk of gastric cancer among individuals (RR, 0.70; 95% CI 0.57–0.87; *I*^2^ = 0%, comparing combined estrogen-progestin therapy vs. non-users) (Fig. 3B).

### Sensitivity analysis and publication bias

In additional analysis, the risk estimate was generally consistent after we reran the meta-analysis with a fixed-effect model (Pooled RR, 0.72; 95% CI 0.64–0.80; *I*^2^ = 0%) (Figure S2). In leave-one-out meta-analysis, no study had a substantial impact on the pooled risk estimate (Supplementary Figure S3). The funnel plot asymmetry (Supplementary Figure S4) and Egger test (*p* = 0.416) of the association between the use of HRT and risk of gastric cancer suggested that there was no publication bias and small-study effects; and therefore, trim-and-fill method was not conducted.

## Discussion

In this study, we used non-overlapping data from ten studies comprising 1,919,089 women to conduct a systematic review and meta-analysis and examine the association between HRT use and the risk of gastric cancer. The pooled results showed that women who used HRT were at a 28% lower risk of gastric cancer, compared to non-users. Estrogen-only therapy was associated with a 37% reduction in gastric cancer incidence and combined estrogen-progestin therapy reduced gastric cancer risk by 30%. The estimations for prediction intervals were in line with the main analysis. In sensitivity analysis, the results did not change substantially when using the fixed-effect meta-analysis.

Our findings are broadly in line with the previous meta-analysis, including seven studies published before 2011^16^. The current meta-analysis incorporated the most updated evidence and conducted stratified analysis according to specific types of HRT, which were not considered in the previous meta-analysis. Our results suggested that both estrogen-only therapy and combined estrogen–progestin therapy were associated with lower gastric cancer incidence, providing new insight to the assessment of benefits and risks among individuals who had been prescribed with estrogen-only HRT after hysterectomy. The prevalence of HRT use has reduced significantly after the Women’s Health Initiative study suggested that HRT use was associated with a number of adverse health outcomes^36^. Despite the fact that HRT remains a management option for women with menopausal symptoms and its benefits may outweigh the harms^37,38^, many may focus on side effects without considering all the available evidence when making choices. Nevertheless, our findings that HRT was associated with a lower risk of gastric cancer does not ipso facto imply that HRT should be prescribed as primary preventive measure. Finally, due to paucity of data, we were unable to perform analysis stratified by dosage and duration. Given that stomach cancer is the seventh most common cancer in women, affecting 1 in 80 women during their lifetime^39^, our findings justify further preclinical research and explorations on the link between HRT use and gastric cancer risk according to the dosage and duration of use.

Although the causal pathway has not been well established, our findings provided evidence that hormone use may lead to a favourable outcome for primary gastric cancer prevention. There are several mechanisms through which exposure to HRT may lead to a reduction of gastric cancer risk. The presence of estrogen receptors-beta (ERβ) have been demonstrated in gastric adenocarcinoma^40^, and exogenous hormone binding could result in inhibition of cancer cell growth and induce apoptosis^41^. The findings of a Korea study using human gastric cancer cell lines implied inhibitory effects of HRT on the ERβ-positive gastric cancer^42^. Indeed, loss of ERβ expression was associated with poor gastric cancer survival^40^. In addition, a selective estrogen receptor modulator was suggested to promote gastric carcinogenesis via antiestrogenic effects in breast cancer survivors^43^. A previous study using data from the Swedish Cancer Registry showed a shorter latency of gastric cancer development in breast cancer survivors who had tamoxifen, as compared with non-users^44^.

To our knowledge, this study is the most updated and comprehensive meta-analysis that examines the association of HRT use with the risk of gastric cancer. The present study is also the first to provide subgroup analyses based on HRT formulation. This meta-analysis has some limitations. First, eight of the 11 included studies used self-reported questionnaires to assess the usage of HRT^18,19,26,29–31,34,35^. However, among these eight studies, five studies were prospective design, which may minimize the bias from misclassification since any misreporting may be random and unrelated to outcomes. Second, observational studies are susceptible to residual confounding. In this analysis, the majority of the included studies adjusted for major risk factors for gastric cancer: cigarette smoking was adjusted in nine out of 11 studies^17–19,26,29–31,33,35^, alcohol consumption was adjusted in five studies^17,26,30,33,35^, and body mass index (BMI) or obesity was adjusted in eight studies^17–19,29–31,33,35^. Third, despite rigorously searching for the literature, only three of 11 studies included were from gastric cancer high incidence countries (China, Japan, and Korea)^26,31,32^. Third, the relatively small cancer case number of the included studies may limit statistical power. Nevertheless, we provided prediction intervals indicating the potential findings of future studies. Fourth, the current available evidence does not allow us to conduct subgroup analysis based on cancer histology, location or *H. pylori* infection status. Given the heterogeneity in gastric cancer, the results of the present study may not represent a specific type of gastric cancer. Lastly, the possibility of health user bias cannot be entirely excluded, in which HRT users may have different lifestyle behaviors from non-users. The adjustment for cigarette smoking, alcohol consumption, and BMI in most included studies may lessen the concerns regarding health user bias. While the current evidence suggests that HRT may be of clinical benefit in the reduction of gastric cancer risk, the findings do not necessarily support HRT use for the purpose of cancer prevention. Further clinical assessment is needed to consider the balance between benefits and harms of HRT use in the setting of chronic disease prevention.

## Conclusion

In conclusion, this meta-analysis of observational studies showed that the use of HRT was associated with a lower risk of gastric cancer regardless of HRT formulation. Further studies are needed to investigate the mechanisms and to explore the associations by various dosage and duration.

## Supplementary Information

Supplementary Information.

## Abbreviations

CI: Confidence intervals
ERß: Estrogen receptor beta
HR: Hazard ratio
HRT: Hormone replacement therapy
NOS: Newcastle–Ottawa scale
OR: Odds ratio
RR: Relative risk
