# Supplementary material for: Association of hormone replacement therapy with risk of gastric cancer: a systematic review and meta-analysis
Source: Sci Rep. 2022 Jul 29;12:12997. doi: 10.1038/s41598-022-17345-2 (PMC9338312; doi:10.1038/s41598-022-17345-2)
Supplement: Supplementary file 1 — Supplementary Information. [file 41598_2022_17345_MOESM1_ESM.docx]

**Association of hormone replacement therapy with risk of gastric cancer: a systematic review and meta-analysis**

***Supplementary Online Content***

**Table S1.** Preferred Reporting Items for Systematic Reviews and Meta-Analyses (PRISMA) guidelines for the reporting of meta-analyses.

**Table S2.** MOOSE (Meta-analyses Of Observational Studies in Epidemiology) Checklist

**Table S3.** Search strategies on PubMed.

**Table S4.** Search strategies on the Ovid Embase.

**Table S5.** Search strategies on the Cochrane library.

**Table S6.** Study characteristics of included studies.

**Table S7.** Newcastle-Ottawa Quality Assessment Scale for cohort studies.

**Table S8.** Newcastle-Ottawa Quality Assessment Scale for case-control studies.

**Figure S1.** Prediction interval of gastric cancer risk for the use of hormone replacement therapy, using random-effects model.

**Figure S2.** Forest plot of risk estimates of gastric cancer risk using the fixed-effect model.

**Figure S3.** Leave-one-out meta-analysis.

**Figure S4.** Funnel plot of gastric cancer risk.

**Table S1.** Preferred Reporting Items for Systematic Reviews and Meta-Analyses (PRISMA) guidelines for the reporting of meta-analyses

| **Section/topic** | **#** | **Checklist item** | **Reported on page #** |
| --- | --- | --- | --- |
| **TITLE** | | |  |
| Title | 1 | Identify the report as a systematic review, meta-analysis, or both. | Title |
| ABSTRACT | | |  |
| Structured summary | 2 | Provide a structured summary including, as applicable: background; objectives; data sources; study eligibility criteria, participants, and interventions; study appraisal and synthesis methods; results; limitations; conclusions and implications of key findings; systematic review registration number. | Abstract |
| **INTRODUCTION** | | |  |
| Rationale | 3 | Describe the rationale for the review in the context of what is already known. | Introduction, paragraph 1 and 2 |
| Objectives | 4 | Provide an explicit statement of questions being addressed with reference to participants, interventions, comparisons, outcomes, and study design (PICOS). | Introduction paragraph 3 |
| **METHODS** | | |  |
| Protocol and registration | 5 | Indicate if a review protocol exists, if and where it can be accessed (e.g., Web address), and, if available, provide registration information including registration number. | Methods, paragraph 1 |
| Eligibility criteria | 6 | Specify study characteristics (e.g., PICOS, length of follow-up) and report characteristics (e.g., years considered, language, publication status) used as criteria for eligibility, giving rationale. | Methods, paragraph 3 |
| Information sources | 7 | Describe all information sources (e.g., databases with dates of coverage, contact with study authors to identify additional studies) in the search and date last searched. | Methods, paragraph 2 |
| Search | 8 | Present full electronic search strategy for at least one database, including any limits used, such that it could be repeated. | Supplementary Table S2-S4 |
| Study selection | 9 | State the process for selecting studies (i.e., screening, eligibility, included in systematic review, and, if applicable, included in the meta-analysis). | Methods, paragraph 2 and 3 |
| Data collection process | 10 | Describe method of data extraction from reports (e.g., piloted forms, independently, in duplicate) and any processes for obtaining and confirming data from investigators. | Methods, paragraphs 4 |
| Data items | 11 | List and define all variables for which data were sought (e.g., PICOS, funding sources) and any assumptions and simplifications made. | Methods, paragraph 4 |
| Risk of bias in individual studies | 12 | Describe methods used for assessing risk of bias of individual studies (including specification of whether this was done at the study or outcome level), and how this information is to be used in any data synthesis. | Methods, paragraph 4 |
| Summary measures | 13 | State the principal summary measures (e.g., risk ratio, difference in means). | Methods, paragraph 5 |
| Synthesis of results | 14 | Describe the methods of handling data and combining results of studies, if done, including measures of consistency (e.g., *I^2^*) for each meta-analysis. | Methods, paragraph 5 |

**Table S2.** MOOSE (Meta-analyses Of Observational Studies in Epidemiology) Checklist

| Item No | Recommendation | Reported on Page No |
| --- | --- | --- |
| Reporting of background should include | | |
| 1 | Problem definition | 4 |
| 2 | Hypothesis statement | 4 |
| 3 | Description of study outcome(s) | 4 |
| 4 | Type of exposure or intervention used | 4 |
| 5 | Type of study designs used | 5 |
| 6 | Study population | 5 |
| Reporting of search strategy should include | | |
| 7 | Qualifications of searchers (eg, librarians and investigators) | 5 |
| 8 | Search strategy, including time period included in the synthesis and key words | 5, Supplementary file 1 |
| 9 | Effort to include all available studies, including contact with authors | 5 |
| 10 | Databases and registries searched | 5 |
| 11 | Search software used, name and version, including special features used (eg, explosion) | n/a |
| 12 | Use of hand searching (eg, reference lists of obtained articles) | 5 |
| 13 | List of citations located and those excluded, including justification | Figure 1 |
| 14 | Method of addressing articles published in languages other than English | 5 |
| 15 | Method of handling abstracts and unpublished studies | 5 |
| 16 | Description of any contact with authors | n/a |
| Reporting of methods should include | | |
| 17 | Description of relevance or appropriateness of studies assembled for assessing the hypothesis to be tested | 5 |
| 18 | Rationale for the selection and coding of data (eg, sound clinical principles or convenience) | 5 |
| 19 | Documentation of how data were classified and coded (eg, multiple raters, blinding and interrater reliability) | 6 |
| 20 | Assessment of confounding (eg, comparability of cases and controls in studies where appropriate) | 6 |
| 21 | Assessment of study quality, including blinding of quality assessors, stratification or regression on possible predictors of study results | 6 |
| 22 | Assessment of heterogeneity | 6 |
| 23 | Description of statistical methods (eg, complete description of fixed or random effects models, justification of whether the chosen models account for predictors of study results, dose-response models, or cumulative meta-analysis) in sufficient detail to be replicated | 6 |
| 24 | Provision of appropriate tables and graphics | Figure 1, Supplementary file 1 |
| Reporting of results should include | | |
| 25 | Graphic summarizing individual study estimates and overall estimate | Figure 2 |
| 26 | Table giving descriptive information for each study included | Table S5 |
| 27 | Results of sensitivity testing (eg, subgroup analysis) | Figure 3, Figure S1, Figure S2 |
| 28 | Indication of statistical uncertainty of findings | 7, 8 |
| Reporting of conclusions should include | | |
| 32 | Consideration of alternative explanations for observed results | 10 |
| 33 | Generalization of the conclusions (i.e. appropriate for the data presented and within the domain of the literature review) | 11 |
| 34 | Guidelines for future research | 11 |
| 35 | Disclosure of funding source | 12 |

**Table S3.** Search strategies on the PubMed

| #1 AND #2 AND #3 (articles = 993) |
| --- |
| #1 Exposure: hormone replacement therapy (articles = 533,268)  *estrogen [Mesh] OR *estrogen [tw] OR *estrogen replacement therapy [Mesh] OR *estrogen replacement therapy [tw] OR hormone replacement therapy [Mesh] OR hormone replacement therapy [tw] OR estradiol [Mesh] OR estradiol [tw] OR progestin therapy [Mesh] OR progestin therapy [tw] OR progestin [Mesh] OR progestin [tw] OR *progesterone [Mesh] OR *progesterone [tw] OR HRT [Mesh] OR HRT [tw] OR tibolone [Mesh] OR tibolone [tw] OR norethisterone [Mesh] OR norethisterone [tw] OR norethindrone [Mesh] OR norethindrone [tw] OR medrogestone [Mesh] OR medrogestone [tw] OR menopausal hormone therapy [Mesh] OR menopausal hormone therapy [tw] OR hormone therapy [Mesh] OR hormone therapy [tw] |
| #2 Outcome: gastric cancer (articles = 439,213)  stomach cancer [MeSH] OR stomach cancer [tw] OR stomach neoplasm* [MeSH] OR stomach neoplasm* [tw] gastric cancer [MeSH] OR gastric cancer [tw] OR gastrointestinal neoplasm [MeSH] OR gastrointestinal neoplasm* [tw] OR stomach adenocarcinoma [MeSH] OR stomach adenocarcinoma* [tw] OR gastric adenocarcinoma [MeSH] OR gastric adenocarcinoma* [tw] OR stomach malignan* [tw] OR gastric malignan* [tw] OR gastrointestinal cancer [MeSH] OR gastrointestinal cancer [tw] |
| #3 Study Design: RCTs, cohort, and case-control (articles = 4,075,232)  (randomised controlled trials [MeSH] OR cohort studies [MeSH] OR longitudinal studies [MeSH] OR follow up studies [MeSH] OR prospective studies [MeSH] OR retrospective studies [MeSH] OR survival analysis [MeSH] OR population-based stud* OR follow-up OR cohort [tw] OR longitudinal [tw] OR prospective [tw] OR retrospective [tw] OR incidence stud* [tw] OR incidence stud* [tw] OR concurrent stud* [tw] OR follow up [tw] OR case control studies [MeSH] OR (case [tw] AND control [tw])) NOT (meta-analysis [MeSH]) |

**Table S4.** Search strategies on the Ovid Embase

| #1 AND #2 AND #3 (articles = 102) |
| --- |
| #1 Exposure: hormone replacement therapy (articles = 219,672)  *estrogen OR *estrogen replacement therapy OR hormone replacement therapy OR estradiol OR progestin therapy OR progestin OR *progesterone OR HRT OR tibolone OR norethisterone OR norethindrone OR medrogestone OR menopausal hormone therapy OR hormone therapy |
| #2 Outcome: gastric cancer (articles = 105,199)  stomach cancer OR stomach neoplasm OR gastric cancer OR gastrointestinal neoplasm OR stomach adenocarcinoma OR gastric adenocarcinoma OR stomach malignan* OR gastric malignan* OR gastrointestinal cancer |
| #3 Study Design: RCTs, cohort, and case-control (articles = 2,825,269)  (randomised controlled trials OR cohort studies OR longitudinal studies OR follow up studies OR prospective studies OR retrospective studies OR survival analysis OR population-based stud* OR follow-up OR cohort OR longitudinal OR prospective OR retrospective OR incidence stud* OR incidence stud* OR concurrent stud* OR follow up OR case control studies) NOT (meta-analysis) |

**Table S5.** Search strategies on the Cochrane library

| #1 AND #2 AND #3 (articles = 0) |
| --- |
| #2 Exposure: hormone replacement therapy (articles = 350)  *estrogen OR *estrogen replacement therapy OR hormone replacement therapy OR estradiol OR progestin therapy OR progestin OR *progesterone OR HRT OR tibolone OR norethisterone OR norethindrone OR medrogestone OR menopausal hormone therapy OR hormone therapy |
| #1 Population: gastric cancer (articles = 307)  stomach cancer OR stomach neoplasm OR gastric cancer OR gastrointestinal neoplasm OR stomach adenocarcinoma OR gastric adenocarcinoma OR stomach malignan* OR gastric malignan* OR gastrointestinal cancer |
| #3 Study Design: RCTs, cohort, and case-control (articles = 5117)  (randomised controlled trials OR cohort studies OR longitudinal studies OR follow up studies OR prospective studies OR retrospective studies OR survival analysis OR population-based stud* OR follow-up OR cohort OR longitudinal OR prospective OR retrospective OR incidence stud* OR incidence stud* OR concurrent stud* OR follow up OR case control studies) NOT (meta-analysis) |

**Table S6.** Study characteristics of included studies

| **First author (year)** | **Country** | **Study design** | **Study period** | **Follow up** | **Number of Participants** | **Number of GC cases** | **Age** | **HRT type** | **HRT Assessment** | **Adjusted variables** |
| --- | --- | --- | --- | --- | --- | --- | --- | --- | --- | --- |
| **Prospective cohort** | | | | | | | | | | |
| Brusselaers  (2017) ^17^ | Sweden | Prospective Cohort | 2005-2012 | Median 7yrs | 1,160,351 | 731 | >40 | E/EPT | The Swedish Prescribed Drug Registry | Matched to age, parity, thrombotic events, hysterectomy, diabetes, obesity, smoking-related diseases and alcohol-related diseases |
| Wang  (2016) ^18^ | Singapore | Prospective Cohort | 1993-2013 | Mean 16yrs | 34,022 | 269 | 45-74 | E/EPT | Questionnaire | Age, baseline interview year, father’s dialect, BMI, educational level, smoking status, daily coffee intake, and sodium intake |
| Duell  (2010) ^29^ | Denmark, France, Germany, Greece, Italy, the Netherlands, Norway, Spain, Sweden, and the UK | Prospective Cohort | 1992-2004 | Mean 8.7yrs | 335,216 | 181 | 35-70 | E/EPT | Questionnaire | Age, center, smoking status, education, BMI, and calorie-adjusted vegetable, fruit, red meat, and processed meat |
| Freedman  (2010) ^30^ | USA | Prospective Cohort | 1995-2003 | Median 7.5yrs | 125,887 | 137 | 50-71 | E/EPT | Questionnaire | Age, BMI, fruit and vegetable consumption, smoking use, alcohol intake, physical activity, and total energy intake. |
| Freedman  (2007) ^31^ | China | Prospective Cohort | 1997-2004 | 419260  person-yrs | 73,442 | 154 | 40-70 | E/EPT | Questionnaire | Age, BMI, education, income, cigarette smoking status and smoking dose |
| Nam  (2021) ^32^ | Korea | Retrospective Cohort | 2002-2013 | Median 6.6yrs | 133,690 | 468 | >40 | E/EPT | Claim data | Age group, income, region, Charlson comorbidity index, and year of study entry |
| Kaneko  (2003) ^26^ | Japan | Prospective Cohort | 1988-1997 | Mean 8.2yrs | 40,535 | na | 40-79 | E/EPT | Questionnaire | Smoking status, family history, past history of peptic ulcer, education, dietary factors: alcohol intake, number of rice bowls, and dietary consumption of meat, green and yellow vegetables, bean-paste soup, and green tea |
| **Case-control** | | | | | | | | | | |
| Lope  (2016) ^19^ | Spain | Case-control | 2008-2013 | na | 3,588 | 111 | 20-85 | E/EPT | Questionnaire | Age, educational level, BMI, family history of gastric or colorectal cancer, and tobacco |
| Green  (2011) ^33^ | United Kingdom | Nested case-control | 1995-2005 | Mean 7.5yrs | 4,472 | 750 | >50 | E/EPT | General Practice Research Database | Smoking status, alcohol intake and BMI |
| Frise,  (2006) ^34^ | Canada | Case-control | 1995-1998 | na | 652 | 326 | 20-74 | E/EPT | Questionnaire | Age |
| Fernandez  (2003) ^35^ | Italy | Case-control | 1983-1999 | na | 7,234 | 258 | 45-79 | E/EPT | Questionnaire | Age, study center, year of interview, education, smoking, drinking, type of menopause, age at menopause and BMI |

BMI, body mass index; EPT, estrogen-progestin therapy; E, estrogen therapy; GC, gastric cancer; HRT, hormone replacement therapy; na, not applicable; yrs, years

**Table S7.** Newcastle-Ottawa Quality Assessment Scale for cohort studies

| **Author** | **Year** | **Selection** |  |  |  | **Comparability** | **Outcome** |  |  | **Total stars** | **Risk of bias** |
| --- | --- | --- | --- | --- | --- | --- | --- | --- | --- | --- | --- |
|  |  | **Representativeness of the exposed cohort** | **Selection of the non-exposed cohort** | **Ascertainment of exposure** | **Demonstration that outcome of interest was not present at start of study** | **Comparability of cohorts on the basis of the design or analysis** | **Assessment of outcome** | **Was follow-up long enough for outcomes to occur** | **Adequacy of follow up of cohorts** |  |  |
| Brusselaers ^17^ | 2017 | * | * | * | * | ** | * | * | * | 9 | low |
| Wang ^18^ | 2016 | * | * |  | * | ** | * | * | * | 8 | low |
| Duell ^29^ | 2010 | * | * |  | * | ** | * | * | * | 8 | low |
| Freedman ^30^ | 2010 | * | * |  | * | ** | * | * | * | 8 | low |
| Freedman ^31^ | 2007 | * | * |  | * | ** | * | * | * | 8 | low |
| Nam ^32^ | 2021 | * | * | * | * | ** | * | * | * | 9 | low |
| Kaneko ^26^ | 2003 | * | * |  | * | ** | * | * | * | 8 | low |

Note. Studies awarded three or four stars for selection, two for comparability, and two or three for ascertainment of the outcome were defined to have low risk of bias. Medium risk of bias defined as those studies that rewarded two stars for selection, and one or two stars for comparability, and two or three for ascertainment of the outcome. Studies with zero or one stars for selection, or zero stars for comparability, or zero or one stars for ascertainment of the outcome were defined to have high risk of bias.

**Table S8.** Newcastle-Ottawa Quality Assessment Scale for case-control studies

| Author | Year | Selection |  |  |  | Comparability | Exposure |  |  | Total stars | Risk of bias |
| --- | --- | --- | --- | --- | --- | --- | --- | --- | --- | --- | --- |
|  |  | **Adequate case definition** | **Cases representativeness** | **Controls selection** | **Controls definition** | **Comparability** | **Ascertainment of exposure** | **Same method of ascertainment for cases and controls** | **Non-response rate** |  |  |
| Lope ^19^ | 2016 | * | * | * | * | ** |  | * |  | 7 | high |
| Green ^33^ | 2011 |  | * | * | * | ** | * | * |  | 7 | low |
| Frise ^34^ | 2006 |  | * | * | * | * |  | * |  | 5 | high |
| Femandez ^35^ | 2003 | * | * |  | * | ** | * | * | * | 8 | low |

Note: Studies awarded three or four stars for selection, two for comparability, and two or three for ascertainment of the outcome were defined to have low risk of bias. Medium risk of bias defined as those studies that rewarded two stars for selection, and one or two stars for comparability, and two or three for ascertainment of the outcome. Studies with zero or one stars for selection, or zero stars for comparability, or zero or one stars for ascertainment of the outcome were defined to have high risk of bias.

**Figure S1.** Prediction interval of gastric cancer risk for the use of hormone replacement therapy, using random-effects model.

**Figure S2.** Forest plot of risk estimates of gastric cancer risk using the fixed-effect model.

**Figure S3.** Leave-one-out meta-analysis.

**Figure S4.** Funnel plot of gastric cancer risk.
